# Infection after open heart surgery in Golestan teaching hospital of Ahvaz, Iran

**DOI:** 10.1016/j.dib.2017.11.046

**Published:** 2017-11-20

**Authors:** Roohangiz Nashibi, Mohammad Javad Mohammadi, Seyed Mohammad Alavi, Farid Yousefi, Shokrolah Salmanzadeh, Fatemeh Ahmadi, Mehran Varnaseri, Asghar Ramazani, Sasan Moogahi

**Affiliations:** a Health Research Institute, Infectious and Tropical Diseases Research Center, Ahvaz Jundishapur University of Medical Sciences, Ahvaz, Iran; b Abadan school of Medical Sciences, Abadan, Iran; c Golestan Teaching Hospital, Clinical Research Development Center, Golestan Hospital, Ahvaz Jundishapur University of Medical Sciences, Ahvaz, Iran

**Keywords:** OHS, open heart surgery, Nis, Nosocomial infections, Infection, Heart surgery, Hospital, Iran

## Abstract

The present study surveyed demographic and infection data which were obtained after open heart surgery (OHS) through patient's admission in Golestan teaching hospital, Ahvaz metropolitan city of Iran, taking into account the confirmed location of the infection, microorganism and antibiotic susceptibility. The occurrence of infection among patients during 48 to 72 h after surgery and hospital admission is the definition of Nosocomial infections (NIs) (Salmanzadeh et al., 2015) [1]. All of them after OHS were chosen for this study. In this paper, type of catheter, fever, type of microorganism, antibiotic susceptibility, location of the infection and outcome (live or death) were studied (Juhl et al., 2017; Salsano et al., 2017) [2], [3]. After the completion of the observations and recording patients' medical records, the coded data were fed into EXCELL. Data analysis was performed using SPSS 16.

**Specifications Table**

**Table:** 

Subject area	*Medicine, clinical research*
More specific subject area	*Infection after open heart surgery*
Type of data	*Table, figure*
How data was acquired	*Functional clinical assessment of the patients after open heart surgery.*
Data format	*Raw, analyzed, descriptive and statistical data*
Experimental factors	– *Sample consisted of patients who were admitted after open heart surgery in Golestan teaching hospital.* – *After open heart surgery, demographic data and infection clinical symptoms were gathered via observations and patients' medical records.* – *In this paper, type of catheter, fever, type of microorganism, antibiotic susceptibility, location of the infection and outcome (live or death) have been studied.*
Experimental features	*Infection is* one of the important factors *endangering patients after open heart surgery.*
Data source location	*Ahvaz, Iran*
Data accessibility	*Data are included in this article.*

**Value of the data**

• These data describe effective factors of infection development after open heart surgery; they are useful for promoting the knowledge of community in order to control and prevent infection after open heart surgery.
• Due to the importance of the risk factors of infection among patients who were admitted after open heart surgery, these factors are discussed in this article.
• The results showed that infection can increase the retention time of hospitalization and death among patients after open heart surgery.
• The results of this study can be used in a prevention program in order to decrease infection among teaching hospitals.
• Results are also important for patients after open heart surgery in order to enhance the care and safety.

## 1 Data

Table 1 represents demographic characteristics of patients of open heart surgery in Golestan teaching hospital in Ahvaz, Iran during 2013–2014. Table 2 shows the data of effective factors of infection after open heart surgery among patients who were admitted to Golestan educational hospital in Ahvaz. The results showed that the most important type of microorganism in patients' infection was related to coagulase negative staphylococci (24.49%). Totally, the most isolated bacteria which cause infections in patients after open heart surgery at Golestan hospital were escherichia coli (22.44%), klebsiella (4.08%), entero bacter (4.08%), streptococci (4.08%), pseudomonas aeruginosa (8.16%), coagulase positive staphylococci (10.2%), coagulase negative staphylococci (24.49%), enterococci (14.29%) and acentobacter (8.16%) (Table 2). Based on the results of this study, among all factors, the highest frequencies were related to the type of microorganism and location of the infection. Factors related to infection were fever (3.33%), location of the infection including (sternum (28.57%), saphenous (12.24%), another organs (59.18%)), and outcome including (live (65%), death (35%)).

**Table 1:** Demographic characteristics of patients open heart surgery.

***Parameter***	***Characteristics***	***Mean***	***Standard deviation***	***Number (In percent)***
Age group	10–29	21	± 1.23	1 (1.67%)
	30–49	38	± 2.74	4 (6.66%)
	50–69	57	± 2.06	45 (75%)
	70–90	75	± 1.58	10 (16.67%)
Sex	Male			26(43.34%)
	Female			34(56.66%)
Underlying disease	Yes			7(11.66%)
	No			53(88.34%)
Immunodeficiency	Yes			0(0%)
	No			60(100%)

**Table 2:** Ranking of factors affecting the created infection after open heart surgery in patients admitted in golestan educational hospital, Ahvaz based on their importance.

**Factors**		***Number***	**Percent**
Fever	–	2	3.33%
Type of microorganism	*Escherichia coli*	11	22.44%
	*Klebsiella*	2	4.08%
	*Entero bacter*	2	4.08%
	*Streptococci*	2	4.08%
	*Pseudomonas aeruginosa*	4	8.16%
	Coagulase positive *staphylococci*	5	10.2%
	Coagulase negative *staphylococci*	12	24.49%
	*Enterococci*	7	14.29%
	*Acentobacter*	4	8.16%
Location of the infection	Sternum	14	28.57%
	Saphenous	6	12.24%
	Another organs	29	59.18%
Outcome	Live	39	65%
	Death	21	35%

## 2 Experimental design, materials and methods

### 2.1 Description of study area

**Fig. 1:**
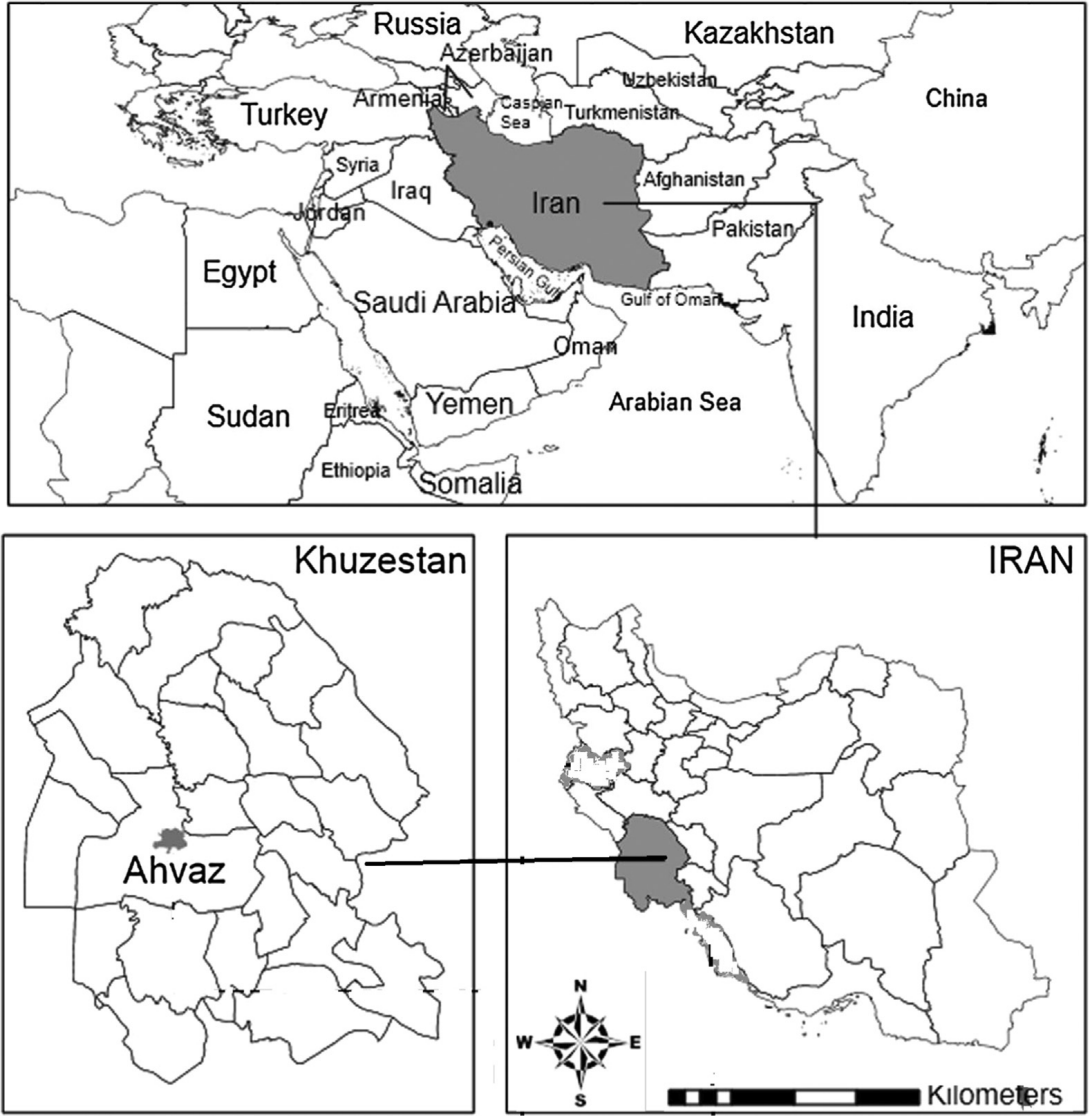
Location of Ahvaz city (*Golestan teaching hospital).*

This cross-sectional study was conducted during 2013–2014 at Golestan teaching hospital of Ahvaz (a tertiary-care hospital) with 450 beds approximately, in the southwest of Iran. Ahvaz, the capital city of Khuzestan province, with an area of 140 square kilometers, is located at 48–49° and 29 min of the eastern longitude in the Greenwich meridian and 30–32° and 45 m of the northern latitude from the equator [4], [5], [6] (see Fig. 1).

### 2.2 Experimental design

For the aims of the study, Golestan teaching hospital was chosen from Ahvaz, Iran. 60 patients who were admitted to Golestan hospital after open heart surgery participated in this study. The gathered data included demographics (e.g. age, sex and underlying disease) and functional clinical assessment of the patients after open heart surgery (including type of catheter, fever, type of microorganism, antibiotic susceptibility, location of the infection and outcome (live or death)).The other sources of data were observations and patients' medical records related to the causes and effective factors of infection after open heart surgery among admitted patients [1], [2], [3], [7]. Then, the coded data were analyzed by SPSS 16 using descriptive statistics. All risk factors of infection were considered as well.
