# Supplementary material for: Infection after open heart surgery in Golestan teaching hospital of Ahvaz, Iran
Source: Data Brief. 2017 Nov 20;16:478–82. doi: 10.1016/j.dib.2017.11.046 (PMC5725203; doi:10.1016/j.dib.2017.11.046)
Supplement: Supplementary file 1 — Transparency document [file mmc1.doc]

**Infection after open heart surgery in Golestan teaching hospital of Ahvaz, Iran**

**Conflicts of Interest**

Authors have no conflicts of interest.

**Acknowledgment**

The authors would like to thank Ahvaz Jundishapur University of Medical Sciences for providing financial supported this research

**Funding/Support**

The authors would like to thank Ahvaz Jundishapur University of Medical Sciences for providing financial supported this research
